# Barriers and solutions for the European prescribing exam: a qualitative world café study

**DOI:** 10.1007/s00228-025-03886-8

**Published:** 2025-07-26

**Authors:** Erik M. Donker, Joost D. Piët, David J. Brinkman, Milan C. Richir, Paraskevi Papaioannidou, Robert Likic, Emilio J. Sanz, Thierry Christiaens, João N. Costa, Fabrizio De Ponti, Milo Gatti, Ylva Böttiger, Cornelis Kramers, Michiel A. van Agtmael, Jelle Tichelaar

**Affiliations:** 1 Department of Internal Medicine, Unit Pharmacotherapy, Amsterdam UMC, Vrije Universiteit, De Boelelaan 1117, Amsterdam, 1081 HV The Netherlands; 2 Research and Expertise Centre in Pharmacotherapy Education (RECIPE), De Boelelaan 1117, Amsterdam, 1081 HV The Netherlands; 3 Department of Surgery, University Medical Center Utrecht, Heidelberglaan 100, Utrecht, 3584 CX The Netherlands; 4 Department of Pharmacology, School of Medicine, Faculty of Health Sciences, Aristotle University of Thessaloniki, University Campus, Thessaloniki, 54124 Greece; 5 Unit of Clinical Pharmacology, Department of Internal Medicine, University Hospital Centre Zagreb and University of Zagreb School of Medicine, 12 Kišpatićeva St, 10 000, Zagreb, Croatia; 6 School of Health Science, Universidad de La Laguna, and Hospital Universitario de Canarias (SCS), Santa Cruz de Tenerife, Calle Padre Herrera, s/n, La Laguna, Santa Cruz de Tenerife, Tenerife, 38200 Spain; 7 Department of Fundamental and Applied Medical Sciences, Unit of Clinical Pharmacology, Ghent University, C. Heymanslaan 10, Ghent, 9000 Belgium; 8 Laboratory of Clinical Pharmacology and Therapeutics, Faculty of Medicine, University of Lisbon, Cidade Universitária, Alameda da Universidade, Lisbon, 1649-004 Portugal; 9 Department of Medical and Surgical Sciences, Pharmacology Unit, Alma Mater Studiorum, University of Bologna, Via Irnerio 48, Bologna, 40126 Italy; 10 Department of Biomedical and Clinical Sciences, Linköping University, Linköping, 581 83 Sweden; 11 Department of Clinical Pharmacy, Canisius Wilhelmina Ziekenhuis, Weg Door Jonkerbos 100, Nijmegen, 6532 SZ The Netherlands; 12 Department of Pharmacy, Pharmacology and Toxicology and Department of Internal Medicine, Radboud University, Geert Grooteplein Zuid 10, Nijmegen, 6525 GA The Netherlands; 13 Interprofessional Collaboration and Medication Safety at the Faculty of Health, Sports and Social Work, Inholland University of Applied Sciences, De Boelelaan 1109, Amsterdam, 1081 HV The Netherlands

**Keywords:** Medical education, Pharmacotherapy, Clinical pharmacology

## Abstract

**Purpose:**

To harmonize and modernize clinical pharmacology and therapeutics (CPT) education across Europe, we developed the European Prescribing Exam. Before its introduction into medical degree programs, it is crucial to understand the potential barriers to its implementation and ways to overcome them. Therefore, the aim of this study was to identify barriers and potential solutions to the implementation of the European Prescribing Exam.

**Methods:**

This qualitative World Café (WC) study involved CPT teachers who participated in a 2-day event focused on the European Prescribing Exam. There were five tables in the WC, each dedicated to a different topic of implementation: (1) organization, (2) technical aspects, (3) content, (4) rollout logistics, and (5) politics. Participants rotated randomly between the tables every 20 min. During each round, they were encouraged to identify barriers and solutions, which were then discussed. The rounds continued until data saturation was reached. Findings were summarized at the end of the WC. We used inductive thematic analysis using a semantic approach to analyze the data.

**Results:**

In total, 26 CPT teachers (female: *n* = 14) from 19 medical schools in 15 European countries participated. After four rounds, 86 potential barriers and 86 solutions were identified. Most barriers were related to the topics “Content” (*n* = 22), “Organization” (*n* = 20), and “Technical aspects” (*n* = 18). Thematic analysis identified 11 themes, three of which were overarching, meaning they applied to multiple topics. The most significant themes included barriers related to curricula, motivation, information technology, and relevance.

**Conclusion:**

This study shows that organizing and implementing the European Prescribing Exam will be challenging. However, participants proposed potential solutions for nearly all barriers, which suggest that the implementation of the European Prescribing Exam is feasible.

**Supplementary Information:**

The online version contains supplementary material available at 10.1007/s00228-025-03886-8.

## Introduction

Although junior doctors are certified to prescribe medicines, they frequently feel unprepared for this task and lack the necessary knowledge and skills for safe and rational prescribing [1–3]. Hence, junior doctors often describe prescribing as challenging, and errors are common [4, 5]. The European Association for Clinical Pharmacology and Therapeutics (EACPT) has recognized this as a significant public health issue and has advocated the need for modernization and harmonization of clinical pharmacology and therapeutics (CPT) education across Europe [6]. Significant milestones in this effort are the consensus reached on the key learning outcomes and the essential diseases that should be covered in CPT education [7, 8]. However, these standards are not yet implemented in all medical schools. Therefore, in 2019, we started the “the European Prescribing Exam,” as part of the Erasmus + project EuroPE^+^, to assess these outcomes in a standardized examination during undergraduate medical training and to promote harmonization across Europe [9].

Five years into this project, we have successfully developed a robust examination platform, three examinations containing 36 (case-based) knowledge questions and 11 prescription questions, and a consensus list of key medicines for prescribing education [10]. The examinations have been piloted in 11 European countries. Recent research underscores the value of a final assessment in improving junior doctors’ prescribing knowledge, highlighting the need for such examinations in all European medical schools [11]. Although the European Prescribing Exam was developed collaboratively by nine medical schools from eight different countries, together with the EACPT and with advisory support from the World Health Organization Europe, reflecting a broad, international perspective, organizing and implementing a pan-European final examination still requires an understanding of potential barriers to the implementation and ways to overcome them. This will guide us in refining and adapting the European Prescribing Exam as needed. The aim of this study was to identify and understand the barriers and potential solutions for implementing the European Prescribing Exam in medical schools throughout Europe.

## Methods

### Study design

**Fig. 1:**
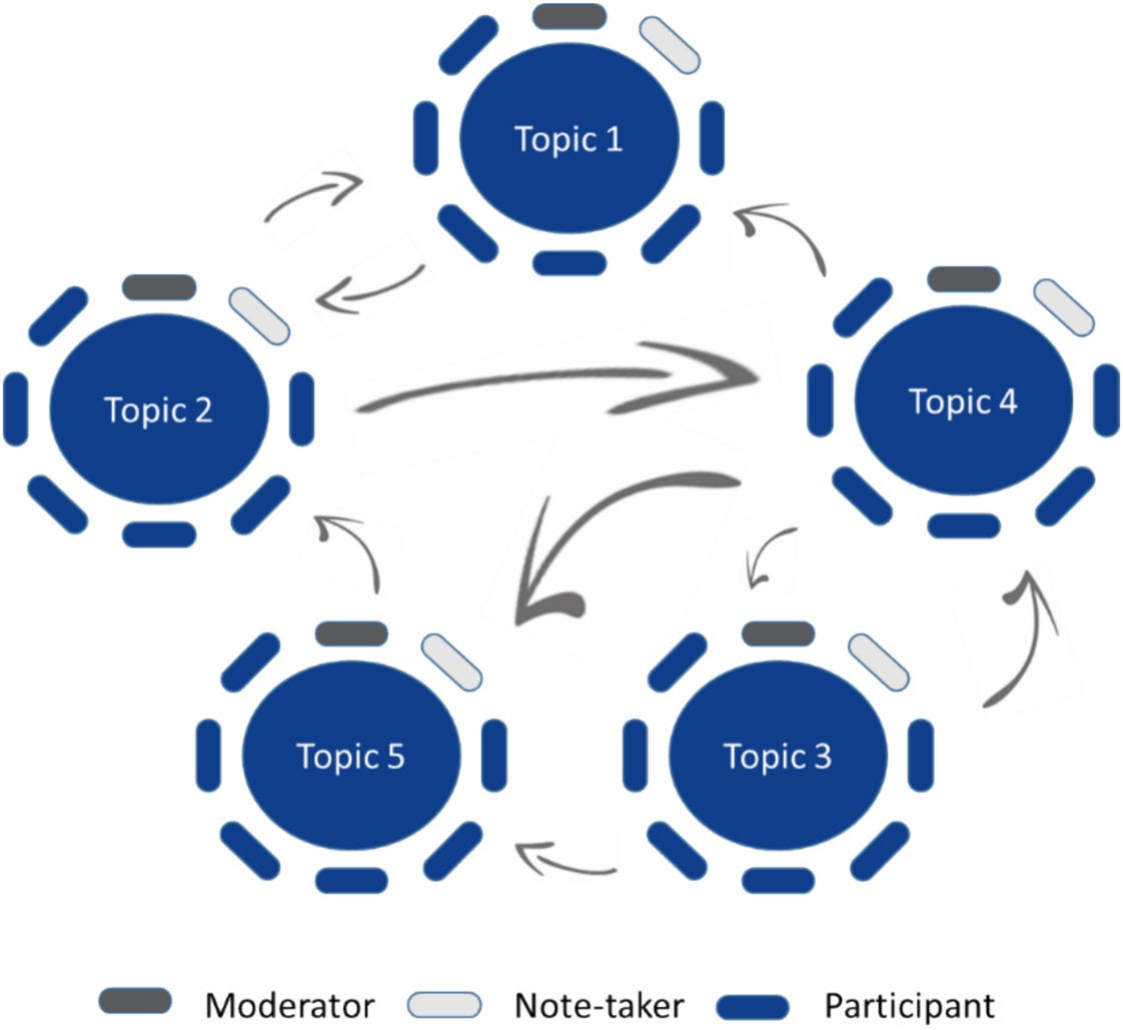
Overview of a World Café

In this qualitative study, we used the World Café (WC) method because of its simplicity and effectiveness in facilitating discussions among large groups. This method is characterized by participatory action research, i.e., collaborative problem solving, with active involvement of all participants, thus promoting diverse perspectives [12]. Typically, a WC involves several rounds during which participants discuss specific topics in different groups. Seven principles are essential to a successful WC: (1) set the context; (2) create hospitable space; (3) explore questions that matter; (4) encourage everyone’s contribution; (5) cross-pollinate and connect diverse perspectives; (6) listen together for patterns, insights, and deeper questions; and (7) harvest and share collective discoveries [12]. The EuroPE^+^ project team, consisting of nine member medical schools from eight countries, identified five key topics essential for implementing the examination. These topics were based on experiences from piloting the European Prescribing Exam and discussions held during project meetings and the EACPT Congress 2022. Each topic was assigned to a separate discussion table. The topics were as follows: (1) organization, focusing on the logistic aspects of the examination; (2) technical aspects, dealing with the technological infrastructure required; (3) content, concerning the subject matter and structure of the examination; (4) rollout logistics, addressing the practical steps needed for rollout; and (5) politics, navigating the regulatory and institutional frameworks. Our WC comprised 20-min rounds until data saturation (no new barriers and/or solutions) was reached. In the initial 2–3 min of each round, participants took time to jot down their perceived barriers and potential solutions related to the topic of the table on a sticky note. Then, everyone shared their input aloud. A member of the research team (ED, DB, JT, FDP, and RL) acted as moderator and a student-assistant from Amsterdam UMC took notes on large flip-overs. After each round, participants were asked to move to a different table at random, but not with the same participants (Fig. 1). When data saturation was reached, there was a debriefing session for all participants, during which the moderators presented a summary of the discussions that had taken place at their respective table. Thereafter, participants could comment on findings and provide additional information or alternative viewpoints, thereby ensuring a comprehensive and inclusive overview of the discussions. For a detailed description of our implementation of the seven steps of a WC, see Appendix 1.

Our WC was held on 10 November 2022 during a 2-day event focused on the European Prescribing Exam at the Vrije Universiteit Amsterdam, the Netherlands. The study was approved by the Medical Ethics Review Committee of Amsterdam University Medical Centers, location Vrije Universiteit (2022.0776).

### Participants

All attendees of the 2-day event about the European Prescribing Exam had been invited and were CPT teachers interested in integrating the European Prescribing Exam in their own medical school. Participation was voluntary; all participants provided their informed consent.

### Data collection and analysis

All flip-overs and sticky notes, without any personal information, were collected for data analysis. For each topic, a list of barriers and solutions was set up. The discussion on these barriers and solutions was analyzed qualitatively, like in other WC studies [13–15], with a view to triangulating the data and providing a deeper understanding of what aspects were deemed important. We used inductive thematic analysis described by Braun and Clarke using a semantic approach to analyze the data with an open perspective and to uncover the explicit meaning of the participants’ statements [16]. This analysis was performed within a realist paradigm. Two researchers (ED and JT) independently performed the coding and discussed the codebook with the research team prior to moving to the next phase of coding. We used the consolidated criteria for reporting qualitative research (COREQ) checklist for interviews and focus groups to report this study [17].

### Research team

As described by Giorgi, thematic analysis requires an open mind to allow unexpected meanings to emerge [18]. However, researchers always bring their own assumptions and beliefs. The five moderators in this WC were white males: four were medical doctors (ED, DB, FdP, and RL), four were clinical pharmacologists (DB, JT, FdP, and RL), one was a clinical pharmacologist in training (ED), and four held a doctorate (DB, JT, FdP, and RL). ED conducted this research as part of his PhD thesis. The moderators were experienced in qualitative research methods such as focus group meetings, interviews, and the nominal group technique. The research team members had met the participants at conferences or previous collaborations. The research group had no financial or professional incentives causing conflict of interest.

## Results

All 26 CPT teachers (female: *n* = 14) from 19 medical schools in 15 European countries (for all countries, see Appendix 2) participated in the study: 19 were clinical pharmacologists (17 medical doctors, 1 pharmacist, and 1 with a bachelor’s degree in medicine; mean working/teaching experience, 13.9 years), 5 were clinical pharmacologists in training (all medical doctors), 1 was a clinical pharmacist, and 1 was a pharmacologist.

### General findings

**Fig. 2:**
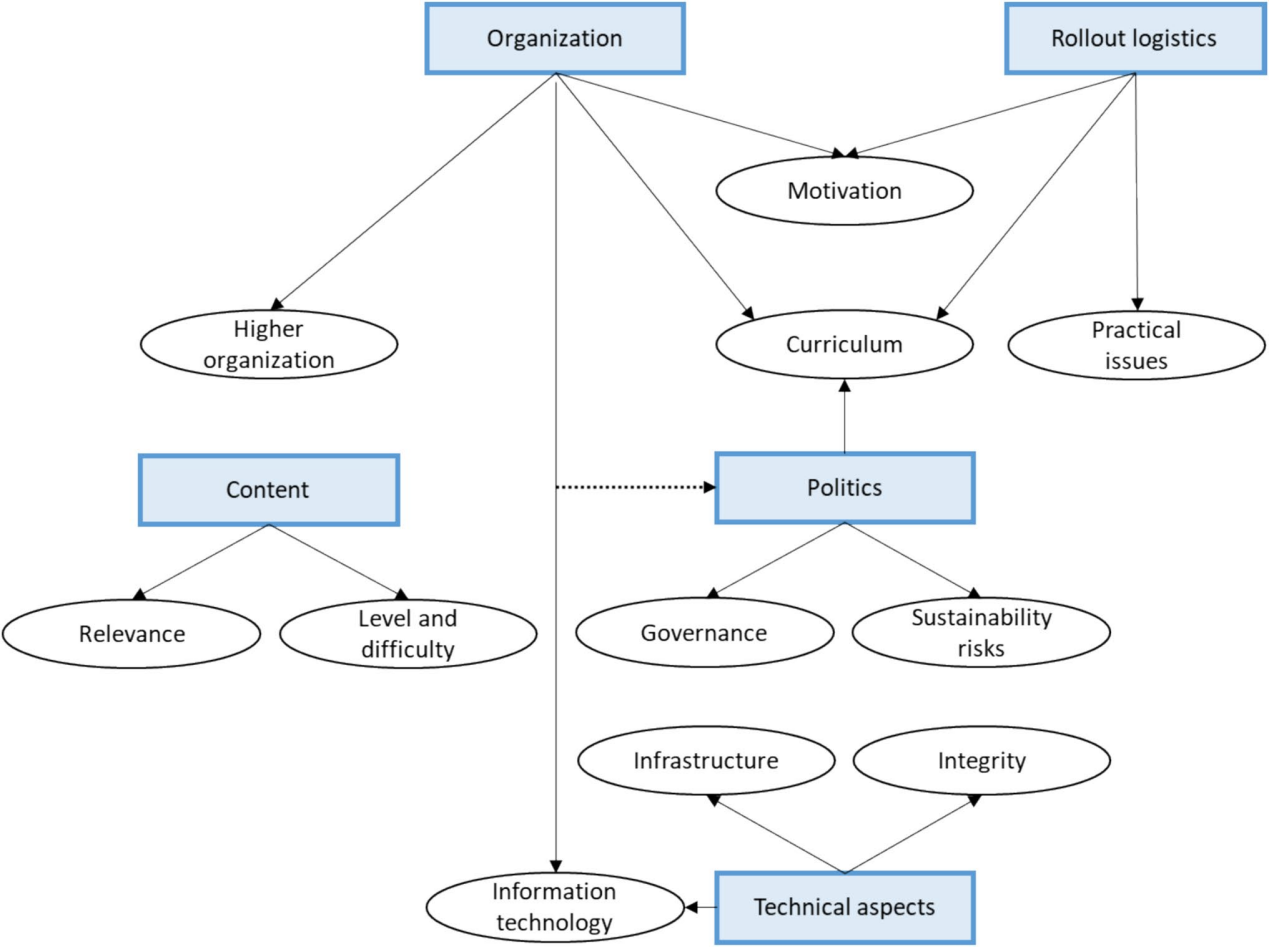
All identified themes. Dotted line: Four barriers identified under the organization topic align with themes from the politics topic

After four 20-min rounds, data saturation was reached, with a total of 86 potential barriers and 86 solutions identified. Most barriers related to the topics “Content” (*n* = 22), “Organization” (*n* = 20), and “Technical aspects” (*n* = 18). Participants did not suggest concrete solutions for seven potential barriers; instead, the research team suggested potential solutions. The discussions at the five tables, each focusing on one of the five topics, led to the identification of 11 themes through thematic analysis (Fig. 2). Some of these themes are overarching, meaning they are relevant to multiple topics. Below, we discuss four themes that emerged prominently during the general debriefing session. A complete overview of all themes, including barriers and proposed solutions, can be found in Appendix 3.

### Curricular barriers and solutions

The participants identified potential curricular barriers for three of the five topics. Existing curricula are overfull, leaving no room for an additional examination or extra teaching hours to prepare students for the European Prescribing Exam. To address this, participants suggested initially introducing the examination as a voluntary/formative assessment or integrating it into existing teaching hours and examinations. However, they raised concerns about the difference in the time allotted to and content of CPT education in European medical curricula. It was pointed out that the European Prescribing Exam was based on European consensus studies and developed by clinical pharmacologists from all participating countries, making it broadly applicable across diverse curricula. Participants also suggested that the European Prescribing Exam could help harmonize CPT education in Europe by providing a standardized examination framework.

From a political perspective, participants noted that integrating the European Prescribing Exam into an existing curriculum could be challenging as curriculum makers may not prioritize changes in CPT education. Additionally, they expressed concern that the use of English might be a barrier for those responsible for its integration, given that the rest of the curriculum is typically in the local language and students’ English proficiency might not be adequate to fully understand the questions. To increase the perceived importance of this initiative, participants suggested presenting curriculum makers with data on the prevalence of prescribing errors, particularly among recently graduated doctors, and with information on the benefits of a final examination on the prescribing skills of students. The issue of language could be addressed by first piloting the examination and by providing information on the benefits of acquiring a high level of English.

### Motivational barriers and solutions

Participants stated that a potential lack of motivation among students, teachers, coordinators, and curriculum makers might be a major barrier to implementing the European Prescribing Exam. All four stakeholder groups must recognize the importance of the examination and be willing to invest in its implementation, which would require time and effort. To overcome this, participants suggested demonstrating the examination’s quality by sharing information about the reliability and validity of the questions. Furthermore, involving students and teachers in the design and formulation of the examination’s questions could improve their understanding of its importance and, consequently, their motivation. Participants also recommended offering training in how to incorporate the examination into curricula, with the added incentive of awarding European Credit Transfer and Accumulation System (ECTS) credits to teachers who complete the training, to further boost teacher participation.

From a political implementation standpoint, participants were concerned that publishing the examination results might expose weaknesses in a medical school’s curriculum and harm its reputation. Therefore, they recommended keeping the examination results confidential, as is currently the practice.

### Information technology (IT) barriers and solutions

Participants identified technological barriers concerning the use of the Moodle platform, which hosts the European Prescribing Exam. The lack of familiarity with Moodle and the difficulty of providing IT support to teachers and students could lead to difficulties in using the system. There was also concern that the system might not adequately support students with disabilities. However, experienced Moodle users confirmed that the platform can accommodate special needs (such as extra time or additional training for students with disabilities). Training sessions on how to use the system could be organized to address potential IT barriers. Furthermore, IT support staff could assist teachers, and comprehensive instruction manuals should be developed for both students and teachers.

Other IT concerns raised were the integrity of the question database and the risk of cheating. However, Moodle is a secure system, making hacking unlikely. In addition, it supports the use of a Safe Exam Browser, which prevents students from accessing other websites during the exam. It was also suggested that examinations be supervised by a teacher or other personnel to further safeguard exam integrity.

### Relevance

Concerns arose about the relevance of the content. Potential barriers to implementing the European Prescribing Exam could arise if the examination primarily focuses on internal medicine and fails to adequately address special situations, such as prescribing during pregnancy, in children, for patients with organ failure, or for the elderly. To address these barriers, participants support the current detailed assessment blueprint, using Miller’s pyramid [19], that includes all critical topics for prescribing. Moreover, they suggested facilitating collaboration with other specialties to ensure that the European Prescribing Exam remains relevant and sustainable. Another concern was the variation in medical guidelines and drug availability and use across different countries, which could complicate the standardization of the examination. The European Key List of Medicines for Medical Education was seen as a positive first step towards harmonization, and participants recommended allowing local adjustments to questions where necessary.

## Discussion

In this WC study, we identified several potential barriers to implementing the European Prescribing Exam. However, participants proposed potential solutions for nearly all these barriers. Nonetheless, it is important to acknowledge that addressing some of these barriers may be challenging and require significant time and effort. The major barriers were related to curricular constraints, motivational challenges, IT infrastructure, and the relevance of the examination. In most countries, curricula are already full and vary widely between institutions, making it difficult to implement a universal examination. Moreover, participants expressed concerns about the language, the time investment, their unfamiliarity with the online system, and the potential impact on medical schools’ reputation if results are published. Despite these concerns, several solutions were proposed. For instance, it was emphasized that the European Prescribing Exam was collaboratively created by clinical pharmacologists from different countries, is based on European consensus studies, and is hosted on a user-friendly online platform. It was suggested that the European Prescribing Exam could initially be used as a formative assessment.

The noted language barrier impacts the foundation of establishing a pan-European examination, as the European Union is characterized by extensive linguistic diversity, with over 20 official languages (e.g., German, French, Spanish). Results from pilot examinations conducted at 16 European universities across 11 countries have shown that language is not a significant barrier. Informal evaluations suggested that students reported that the language did not hinder their understanding or ability to answer the examination questions. These findings support the feasibility of using English as the examination language across different countries. Moreover, advancements in artificial intelligence are making it increasingly easier to translate examination questions accurately in the near future.

Participants also suggested that the final results should not be shared publicly. We noticed a general sense of anxiety among the teachers that students might perform badly on the examination. Many medical schools in Europe still teach their students pharmacology and do not teach them prescribing [20]. The skills part of the European Prescribing Exam focuses on this aspect of CPT and requires students to prescribe for patient cases. We believe the European Prescribing Exam could serve both as a benchmark for proficiency levels that medical graduates should achieve and as a reflective tool for teachers to critically assess their CPT curriculum. The aim is to enable European medical schools to meet the standard of teaching and training in CPT established collaboratively with this examination, not to judge individual schools on their quality.

Another major barrier is that medical curricula are already packed, leaving no room for additional teaching hours or extra examinations. A proposed solution involves using the European Prescribing Exam as a formative assessment, which effectively serves as a first step to convince students, teachers, and curriculum makers of its importance and quality. Formative assessment can also stimulate self-regulated learning by serving as a benchmark for students’ self-reflection [21]. This approach may motivate students to engage more actively in learning and practicing prescribing skills and encourage teachers to adapt the curriculum. Thus, even without a summative component, the European Prescribing Exam has the potential to harmonize CPT teaching and training across Europe. Moreover, in the Netherlands, students recognize the importance of having a dedicated examination on clinical pharmacology, the Dutch National Pharmacotherapy Assessment (DNPA), which facilitates its implementation [22]. If European students also recognize the importance of such an examination, it would facilitate implementation. On the other hand, using the European Prescribing Exam as a formative assessment may also raise questions about its effectiveness. In general, but also in CPT, research shows that scores are lower for formative assessments than for summative assessments because of the lack of (extrinsic) motivation [23, 24]. This was recently confirmed for the DNPA, where scores were significantly higher in medical schools with either a summative or programmatic assessment program rather than a formative assessment program [25]. Programmatic assessment led to a modest decline in scores compared to summative assessment. Therefore, one could argue that the European Prescribing Exam should be incorporated as at least a data point in a programmatic assessment program, with the ultimate goal of implementing it as a summative assessment. On the other hand, incorporating a formative examination, including feedback on provided right and wrong answers in a programmatic assessment curriculum, could well increase the prescribing skills of students without a score.

The IT infrastructure supporting the examination also presents a potential barrier, particularly regarding the unfamiliarity of both teachers and students with the Moodle platform that hosts the European Prescribing Exam. A major advantage is that the Moodle system is accessible on any electronic device with internet access and features an intuitive interface. The Erasmus + project “Clinical Pharmacology and Therapeutics Teach the Teacher (CP4T)” program will organize an online workshop for teachers to improve relevant IT skills. Moreover, Moodle has sufficient database capacity to store all examination questions, addressing one of the potential barriers for which no solution had previously been suggested.

Some barriers were not identified during the WC but were proposed by the authors themselves. One such concern is the sustainability of the European Prescribing Exam, as it was initially funded by an Erasmus + grant that ended in 2023. Currently, the Education Working Group of the EACPT oversees the organization of the exam. Participating medical schools contribute by actively engaging in the development, review, and updating of exam questions.

From a political perspective, concerns may also arise regarding data protection and the security of cloud-based systems. To address this, the European Prescribing Exam is hosted on a European server, and official legal agreements are in place between participating medical schools, the hosting provider, and an intermediary organization.

### Strengths and limitations

This is the first study to investigate potential barriers and solutions for implementing a pan-European examination, as proposed by CPT teachers. The WC method is particularly suitable to investigate this, as it fosters large group discussions and actively involves all participants. Moreover, it aims to find solutions for specific problems or barriers. Also, participants come into contact with their peers and can use the suggested solutions directly after the session. Moreover, this is the first study to explore the potential barriers and solutions for implementing a pan-European examination. Similar examinations have been developed by the European Union of Medical Specialists and the British Pharmacological Society and UK Medical Schools Council (the Prescribing Safety Assessment), but, to our knowledge, these were not evaluated with this method [26, 27]. Nevertheless, some limitations should be taken into consideration when interpreting the results of this study. First, only participants who attended the 2-day event about the European Prescribing Exam participated in this study. This could have biased the results because all participants were intrinsically motivated to implement the examination. On the other hand, this led to positive discussions, and it was a large group of relevant coordinators in CPT education across Europe. Second, besides being notetakers, students did not actively participate in this study, even though they are important stakeholders in examinations. This might have led to missing important barriers and solutions. Third, the project team identified the five topics in advance, so there was no room to discuss other aspects. Moreover, participants might interpret the five topics differently. However, at the start of each round, the moderator introduced the topic, and during the general discussion afterwards, no one added new topics. Fourth, the session was not recorded, so the analysis was only based on the sticky notes, flip-overs, and the interpretation of the moderators and the notetakers afterwards.

### Recommendations

On the basis of findings, we make recommendations for both teachers and the future organization of the European Prescribing Exam. For teachers, (1) as (sometimes) with prescribing, start low and go slow. Curriculum adjustments are challenging and take time. Therefore, we recommend introducing the European Prescribing Exam gradually, initially as a formative examination, and then explore further possibilities. (2) Join the working group for the European Prescribing Exam to ensure the content is relevant to and applicable for your own country. (3) Show both students and curriculum makers the importance of a dedicated examination on CPT that assesses both knowledge and skills to enhance their (intrinsic) motivation. For the future organization, (1) each participating country should appoint a representative to advocate that country’s interests. (2) Each university should contribute to the development of examination questions to ensure broad coverage. (3) Courses should be organized on how the system works and how to manage it effectively. (4) To ensure that the European Prescribing Exam continues to function and evolve in the long term, its sustainability must be regularly reviewed.

## Conclusion

This study shows that organizing and implementing the European Prescribing Exam will be challenging. However, participants proposed potential solutions for nearly all barriers, primarily by introducing the examination gradually and in a formative manner while collaboratively working towards long-term implementation. Future research should explore the feasibility of implementing the European Prescribing Exam in terms of quality, including aspects such as reliability and validity.

## Supplementary Information

Below is the link to the electronic supplementary material.

ESM 1 (DOX 15.7 KB)

ESM 2 (DOCX 12.6 KB)

ESM 3 (DOCX 42.9)

## Data Availability

The data that support the findings of this study are available directly after publication from the corresponding author upon reasonable request.
