# Supplementary material for: Barriers and solutions for the European prescribing exam: a qualitative world café study
Source: Eur J Clin Pharmacol. 2025 Jul 26;81(10):1451–9. doi: 10.1007/s00228-025-03886-8 (PMC12443867; doi:10.1007/s00228-025-03886-8)
Supplement: Supplementary file 1 — (DOX 15.7 KB) [file 228_2025_3886_MOESM1_ESM.docx]

# Appendix 1. A detailed overview of our implementation of the seven steps in the World Café approach

1. Set the context

Before the start of the WC, one of the researchers (ED) explained the procedure (Figure 1), emphasizing that the goal was not to reach consensus but to collect information about as many potential barriers and solutions as possible. Everybody’s opinion was relevant.

1. Create hospitable space

The WC was held in a foyer, with five tables with each a maximum of eight participants. Participants introduced themselves in the first morning during the two-day event. Refreshments were provided during the WC.

1. Explore questions that matter

The EuroPE^+^ project team identified five key topics central to implementation of the examination, with each topic being allocated a separate discussion table. The topics were: (1) organization, focusing on the logistic aspects of the examination; (2) technical aspects, dealing with the technological infrastructure required; (3) content, concerning the subject matter and structure of the examination; (4) implementation, addressing the practical steps needed for rollout; and (5) politics, which involved navigating the regulatory and institutional frameworks.

1. Encourage everyone’s contributions

Our WC comprised four 20-minute rounds. In the initial 2-3 minutes of each round, participants took time to jot down their perceived barriers and potential solutions related to the topic of the table on a sticky note. Then everyone shared their input aloud. A member of our research team (ED, DB, JT, FDP, and RL) acted as moderator and a student-assistant from Amsterdam UMC took notes. Their roles were to guide and note the discussion and ensure that every participant had the opportunity to contribute.

1. Cross-pollinate and connect diverse perspectives

After each round, participants were asked to move to a different table at random, but not with the same participants. At the new table, the moderator briefly presented the barriers and solutions identified by the previous group(s). Then, the new group of participants had 2-3 minutes to note down their own thoughts before the discussion recommenced. This process allowed for a fresh exchange of ideas and perspectives in each round.

1. Listen together for patterns, insights, and deeper questions

The moderators guided the discussions, delving deeper into the participants' responses with probing questions. Large flip-over charts were used to record the barriers and solutions identified by participants. This method facilitated the linking of specific barriers to corresponding solutions and enabled the addition or refinement of ideas.

1. Harvest and share collective discoveries

After four rounds, there was a debriefing session for participants, during which the moderators presented a summary of the discussions that had taken place at their respective table. Thereafter, participants could comment on findings and provide additional information or alternative viewpoints, thereby ensuring a comprehensive and inclusive overview of the discussions.
