# Supplementary material for: Barriers and solutions for the European prescribing exam: a qualitative world café study
Source: Eur J Clin Pharmacol. 2025 Jul 26;81(10):1451–9. doi: 10.1007/s00228-025-03886-8 (PMC12443867; doi:10.1007/s00228-025-03886-8)
Supplement: Supplementary file 2 — (DOCX 12.6 KB) [file 228_2025_3886_MOESM2_ESM.docx]

# Appendix 2. Number of participants per countries

Belgium (n=2)

Bulgaria (n=1)

Croatia (n=2)

Czech Republic (n=2)

Finland (n=1)

France (n=4)

Greece (n=1)

Ireland (n=1)

Italy (n=1)

Malta (n=1)

Portugal (n=1)

Serbia (n=2)

Spain (n=2)

Portugal (n=1)

Sweden (n=1)

The Netherlands (n=3)
