# Supplementary material for: Barriers and solutions for the European prescribing exam: a qualitative world café study
Source: Eur J Clin Pharmacol. 2025 Jul 26;81(10):1451–9. doi: 10.1007/s00228-025-03886-8 (PMC12443867; doi:10.1007/s00228-025-03886-8)
Supplement: Supplementary file 3 — (DOCX 42.9) [file 228_2025_3886_MOESM3_ESM.docx]

Appendix 3. Overview of all barriers and solutions

1. **Organisation (20 barriers/16 solutions)**
   1. Curriculum (7 barriers – 5 solutions)
   2. Motivation (5 barriers – 6 solutions)
   3. Information technology (2 barriers – 2 solutions)
   4. Higher organization (2 barriers – 3 solutions)
   5. Miscellaneous (4 barriers – 1 solutions)
2. **Technical aspects (18 barriers/19 solutions)**
   1. Infrastructure (11 barriers – 6 solutions)
   2. Integrity (3 barriers – 7 solutions)
   3. Information technology (4 barriers – 10 solutions)
3. **Content (22 barriers/16 solutions)**
   1. Relevance (14 barriers – 14 solutions)
   2. Leve and difficulty (8 barriers – 9 solutions)
4. **Rollout logistics (13 barriers/18 solutions)**
   1. Curriculum (5 barriers – 4 solutions)
   2. Motivation (4 barriers – 8 solutions)
   3. Practical issues (3 barriers – 4 solutions)
5. **Politics (13 barriers/16 solutions)**
   1. Curriculum (4 barriers – 8 solutions)
   2. Governance (4 barriers – 7 solutions)
   3. Sustainability risks (5 barriers – 6 solutions)

1 general solution: Write letter of recommendations by EuroPE^+^ on how to implement

## Organization

| Theme of barriers | Barriers | Solutions |
| --- | --- | --- |
| Curriculum | No space in medical curriculum | Start as a voluntary exam |
|  |  | Incorporate in existing EU exam (final year of master) |
|  |  | Harmonization of drug list |
|  | Lack of time to teach and train students appropriately | Combine preparations with existing workshops/training |
|  |  | Add a course/seminar on prescribing |
|  | Lack of flexibility in the timing of the exam | Discuss the moment when to assess with students/medical board |
|  |  | Added by the authors  Leave the decision on timing of assessment to medical schools, not on a national or EU level |
|  | The time and effort required by students | Give students an active role in developing content/evaluating the exam |
|  |  | Show students their progress |
|  | Lack of personnel (teachers/clinical pharmacologists) to adjust the questions to county-specific questions | Combine preparations with already existing workshops/training |
|  | It is difficult to make it a mandatory assessment. | Start as a voluntary exam |
|  | The teaching of pharmacotherapy takes place at different stages (4th, 5th, 6th year) of the medical curriculum. | Discuss the moment when to assess with students/medical board |
| Motivation | Lack of motivation/participation of students | Start as a voluntary exam |
|  |  | Give students an active role in developing content/evaluating the exam |
|  |  | Show students their progress |
|  | Lack of belief in importance among clinicians/directors/students | Universities wishing to use the exam should develop questions |
|  |  | Make it competitive with other schools/tell the European story |
|  | Lack of motivation among CPT teachers | Provide teachers with training |
|  | Lack of ‘believers’ in the importance of exam | From politics table:  Engage other professionals (pharmacists, nurses) to create and participate in the exam.  Work together with other specialties  Stimulate collaboration |
|  | Teachers of other specialties questioning the relevance and need for this exam |  |
| Information technology | Lack of IT specialist | Include IT personnel |
|  | How to manage students with disabilities | Provide extra time/training for students with disabilities |
| Higher organization | Unclear who is responsible (pharmacologists, clinical pharmacologists, clinicians, education board) and what the management structure is | Collaborate with other medical education associations (AMEE, IUPHAR, WHO) |
|  |  | Top-down (EMA or EU 🡪 Countries 🡪 Universities |
|  |  | Hire a project manager to implement it |
|  | Lack of money in EACPT | Added by the authors  Apply for grants  Ask all participating medical schools for compensation (will be low as Moodle’s hosting costs are low, and the project is non-profit) |
| Miscellaneous | Difficult to compare results | Make it competitive with other schools/tell the European story |
|  | Making the exam sustainable in the long term | From politics table:  Engage other professionals (pharmacists, nurses) to create and participate in the exam.  Work together with other specialties  Stimulate collaboration |
|  | No collaboration between universities |  |
|  | Lack of patient involvement | From politics table:  Involve patient organizations |

## Technical aspects

| Theme of barriers | Barriers | Solutions |
| --- | --- | --- |
| Infrastructure | Platform bandwidth and internet requirements | Run multiple sessions to accommodate large numbers of students |
|  | Sustainability of the database (enough questions) | Continuous contribution of questions |
|  | Unclear which resources are allowed | Define allowed resources |
|  | Battery life/lack of power for computers | Warn to charge before the start of the exam |
|  | The dosage options are not always apparent to students | Provide students with dosage options prior to the exam |
|  | Unclear whether personal notes are allowed | Define allowed resources |
|  | Language of the exam and teaching materials | From content table:  Correct language per location |
|  | Are additional resources integrated in the system | Define allowed resources |
|  | Capacity of the question database | Added by the authors  Moodle has sufficient capacity on its database to store all exam questions |
|  | Transition between knowledge and skills parts (do all students have to finish the knowledge part first?) | Added by the authors  Student are free to go to the next part |
|  | Equipment of universities | Added by the authors  Moodle is accessible on all devices |
| Integrity | Protection of the database with exam questions | Create a separate database with exam questions outside Moodle |
|  |  | Use standard database protection |
|  | Cheating | On-site exams supervised by teachers or other personnel |
|  |  | Webcams for online exams |
|  |  | Severe punishment for cheating |
|  |  | Randomization of questions and answers |
|  | Fidelity (teaching) | Teach the teacher |
| Information Technology | Teachers' lack of familiarity with Moodle | Teach the teacher |
|  |  | Create a clear PDF file with instructions |
|  |  | Collect experiences/feedback, and share |
|  | Compatibility of the Moodle system | IT department |
|  |  | Train IT department |
|  |  | Pilot the system with students before the exam (with IT staff) |
|  |  | Collect experiences/feedback, and share |
|  | Need for technical support | IT department |
|  | Lack of compatibility between IT departments | IT department |
|  |  | Train IT department |

## Content

| Theme of barriers | Barriers | Solutions |
| --- | --- | --- |
| Relevance | Most questions are related to internal medicine | More variety in cases/questions (review questions in skills) |
|  | Limited coverage of medicines/topics | Questions on calculations, information etc. |
|  |  | Extend the blueprint (pregnancy/lactation, children, organ failure, elderly) |
|  |  | More variety in cases /questions (review questions in skills) |
|  | Only questions about severe or most common medicines (SAE/ADR) | Extend the blueprint (pregnancy/lactation, children, organ failure, elderly) |
|  | Not enough collaboration with pharmacologists and clinicians, and not interprofessional (nurses) | Encourage collaboration (teaching together, more discussion in committees) |
|  | Lack of student voice in content and design | Give students a voice in content and blueprint |
|  | Availability of medicines differs among countries (and updating the list of essential medicines) | Create a list of ‘group medicines’, instead of individual drugs |
|  | Differences between countries in what is considered basic knowledge/skills for junior doctors and which medicines junior doctors should be able to prescribe | Identify differences between countries |
|  |  | Include more correct answers (teacher can change questions where this is the case before giving the exam) |
|  | Foreign language (English) | Correct language per location |
|  | Update list of essential medicines (enough questions based on this list alone) | Evaluate frequently (review board with physicians and pharmacologists) |
|  | Different levels of knowledge required per topic per curriculum | Identify differences between countries |
|  |  | Evaluate frequently (review board with doctor and pharmacologists) |
|  | Validation of the exam in each language | Correct language per location |
|  | Different use of medicines between countries (e.g. antidepressants). | From organization table:  Harmonization of drug list |
|  | Differences in guidelines between first and second line | From politics table:  Engage other professionals (pharmacists, nurses) to create and participate in the exam.  Work together with other specialties  Stimulate collaboration |
|  | Differences in prescribing habits (1st line, 2nd line, 3rd line) |  |
| Level and difficulty | Real patient cases are complex | Use cases from existing databases |
|  | Unclear what the desired level of skills is (competence) | Adapt cases to the desired level |
|  | Pharmacotherapy education is currently given in different years in different medical schools and in different countries | Adapt cases to the desired level |
|  | The exam only assesses level of knowledge and skills at end of the study | Collect syllabi 🡪 determine best time for exam, or the medical school itself determines when the exam takes place |
|  |  | Adapt cases to the desired level |
|  |  | Monitor students’ progress during their studies with exams between |
|  | Determine the grade or pass/fail threshold? | Each medical school can determine the pass threshold |
|  | Create patient cases for skills | Ask practising physicians for (anonymzed) real cases |
|  |  | Use cases from existing databases |
|  | Having enough questions so that the exam is different each year | From technical aspect and implementation tables:  Continuous contribution of questions  A large pool of questions to prescribe |
|  | Determine desired level and balance between easy and difficult questions (experts create questions for students) |  |

## Rollout logistics

| Theme of barriers | Barriers | Solutions |
| --- | --- | --- |
| Curriculum | Different types of education between universities | Harmonize the learning objectives across the European countries |
|  |  | Build consensus at national level for implementation |
|  | Clinical pharmacology lacks identity in the curriculum | Find partners to help with implementation, as clinical pharmacology is small (but, the more specialists involved, the more discussion) |
|  | The moment in the curriculum | Give the exam to postgraduate students |
|  | Foreign language (English) | From content table:  Correct language per location |
|  | Not all universities spend the same number of hours | Harmonize the learning objectives across the European countries |
|  | What should the pass rate be? Same for all or locally determined | From content table:  Each medical school can determine the pass threshold |
| Motivation | Extra time for teachers | Convince students and staff of the usefulness of the exam (reliability and validity) |
|  | Teachers need to be motivated and inspired | Convince students and staff of the usefulness of the exam (reliability and validity) |
|  |  | Convince on local and national level |
|  |  | National training to discuss and harmonize |
|  | Students do not see the added value of another (extra) exam | Start early with training and preparation of students, mention in lectures |
|  |  | Convince students and staff of the usefulness of the exam (reliability and validity) |
|  |  | Motivate them early so that they see the added value of extra points |
|  |  | Students can tell each other about the value of the exam |
|  | Universities that anticipate poor exam results may not want to implement it | Keep exam results confidential |
| Practical issues | Modest number of questions | Create a large pool of questions |
|  |  | Keep the exam small and accessible at all times so that students can test themselves in a formative way. |
|  | Not having the power to implement the exam | Convince on local and national level |
|  |  | Find a way to justify the extra time spend on clinical pharmacology |
|  | Human resources, IT rooms/facilities | Added by the authors  Moodle is accessible on all devices |

## Politics

| Theme of barriers | Barriers | Solutions |
| --- | --- | --- |
| Curriculum | The exam has no priority in the faculty | Show prescribing and patient safety data |
|  |  | Show the practical benefits for students |
|  | Different curricula/tracks | Look for examples from other faculties |
|  | Foreign language (English) | Show the practical benefits for students |
|  | Lack of collaboration between universities (EU and local) | Harmonize curricula and European drug list for teaching |
|  |  | Stimulate collaboration |
|  |  | Apply for EU funds / grants |
| Governance | Governance | Make the exam obligatory in Europe |
|  |  | Create universal acceptance |
|  | Accreditation body | Top-down strategy (EMA, EU) |
|  | Acceptance in other countries | Top-down strategy (EMA, EU) |
|  |  | Show hard data on patient safety per country |
|  |  | Focus on patient safety |
|  | Lack of awareness in government | Create good marketing idea |
| Sustainability risks | Competition with Prescribing Safety Assessment | Apply for EU funds / grants |
|  | Lack of patient involvement | Involve patient organizations |
|  | Need for visible advocate | Visible advocate |
|  | Lack of professional diversity | Engage other professionals (pharmacists, nurses) to create and participate in the exam. |
|  |  | Work together with other specialties |
|  |  | Stimulate collaboration |
